# Relationship between body mass index and physical fitness in Italian prepubertal schoolchildren

**DOI:** 10.1371/journal.pone.0233362

**Published:** 2020-05-22

**Authors:** Federica Fiori, Giulia Bravo, Maria Parpinel, Giovanni Messina, Rita Malavolta, Stefano Lazzer

**Affiliations:** 1 Department of Medicine, University of Udine, Udine, Italy; 2 Regional School of Sport, Italian Olympic Committee, Trieste, Italy; 3 Regional Italian Olympic Committee, Trieste, Italy; 4 School of Sport Sciences, University of Udine, Udine, Italy; University of Oslo, NORWAY

## Abstract

The objective of this study was to investigate the association between physical fitness and body mass index categories (obesity, OB; overweight, OW; normal-weight, NW; and underweight, UW) in prepubertal children. Anthropometric and physical fitness characteristics were collected from a convenience sample of 30472 Italian schoolchildren (6–11 years old). Six field-based tests were used: Léger, agility shuttle, long jump, frontal throw of the basketball, Sit & Reach and standing balance. Significant differences were found in the anthropometric characteristics, physical fitness and weight status prevalence between girls and boys (p<0.05) and, except for flexibility, by age class (p<0.05). Obese children performed worse than their NW counterparts in aerobic capacity (p<0.001), agility (p<0.001), muscular power of the lower limb (p<0.001) and balance (p<0.001). Conversely, children with obesity showed greater upper limb power than NW children (p<0.001). The discrepancy in physical fitness between OB and NW children increased in older girls (flexibility, p = 0.002; muscular power of the lower and upper limb, p = 0.002 and p = 0.005) and boys (aerobic capacity, p = 0.009; agility, p = 0.006; standing balance, p = 0.019; muscular power of the lower and upper limb, p<0.001 and p = 0.011) compared to their younger counterparts. On the other hand, UW children performed worse than NW children mainly in terms of muscular power of the arms (p<0.001). Additionally, there was an increasing disparity in the frontal throw test scores of UW and NW girls (p = 0.003) and boys (p = 0.011) in older children compared to younger children. In conclusion, the effect of body mass index on children’s physical fitness intensifies with age. OB and OW negatively affect aerobic capacity, agility, lower limb power and balance but positively affect upper limb power. UW negatively affects upper limb power. This study underscores the importance of preventing childhood OW, OB, and UW in early life to promote children’s health and proper fitness development.

## Introduction

In the past four decades, the prevalence of childhood overweight and obesity has risen dramatically worldwide [1]. Italy has one of the highest prevalence of obese and overweight youth in Europe although a trend towards a reduction was observed between 2008 and 2016 [2–4]. Paediatric obesity has a wide range of serious short and long-term health and social consequences. Because of their excess adiposity, obese children may exhibit early signs of multiple comorbidities such as cardiovascular dysfunction [5,6] and asthma [7]. Other short-term consequences of childhood obesity may be underachievement at school, lower self-esteem, psychological problems and decreased quality of life relative to healthy peers [8]. Furthermore, obesity tracks strongly from childhood into later life [9], causing an increased likelihood of morbidity in adulthood as well as all-cause mortality [5].

Another strong predictor of future health is physical fitness, which has been defined as a set of attributes related to a person’s ability to perform physical activities [10]. In the literature, cardiorespiratory fitness and muscular strength have been associated in children with cardio-metabolic risk factors [11,12] and recently, cardiovascular fitness has been positively associated with academic performance [13]. Therefore, physical fitness, and in particular cardiorespiratory fitness, could modify the impact that body mass index (BMI) has on the risk of cardiovascular disease and obesity-related comorbidities, especially in children and adolescents [14,15]. Moreover, research has found positive associations between children’s adiposity and physical fitness, regardless of physical activity levels [10]. In particular, several authors have linked higher BMIs to poor performance in weight bearing activities, i.e., activities that require moving bodyweight through space [16–25], while inconsistences have been found in the association between children’s weight status and muscular strength [18,20,21,23–25]. Given the connections that are present among fitness, weight and health status, monitoring physical fitness levels in prepubertal children may be essential to prevent fitness deficiencies, obesity and their possible health-related consequences [25]. Prepuberty can in fact be a critical time to promote healthy lifestyles in childhood and later life, and the school environment in particular may be suitable for intervention and monitoring programmes due to its capability to reach children across age groups.

After a preliminary evaluation of age- and sex-specific differences in prepubertal children’s physical fitness, the primary purpose of the present study was to analyse the association between physical fitness (cardio respiratory fitness, speed, strength, balance and flexibility) and BMI categories (obesity, OB; overweight, OW; normal-weight, NW; and underweight, UW) in a large sample of Italian schoolchildren. The secondary purpose was to examine whether different physical fitness components improve as a function of age in groups of children of differing weight status. We hypothesized that an OW and OB status in children has a negative effect on physical performance and that such effects increase as a function of age class.

## Subjects and methods

A convenience sample of 30472 Italian schoolchildren (6–11 years old) who participated in the project “*MOVIMENTO in 3S*: *promozione della Salute nelle Scuole attraverso lo Sport*” (*MOVIMENTO in 3S* project: promoting Health in Schools through Sport) was enrolled from different public schools in the Friuli Venezia-Giulia Region (Italy) during the period between 2016 and 2018. The experimental protocol was approved by the University of Udine Ethics Committee on Human Research for Medical Science. The following criteria were adopted to select eligible children: elementary school attendance and the absence of any disease or disability that could make a child unable to participate in the scheduled school physical education programme. Before the study began, the purpose and objectives were carefully explained to each child and his or her parents. Children gave their verbal consent, and written informed consent was obtained from their parents. Then, anthropometric measurements and physical fitness parameters were recorded at the beginning of the school year during school hours. The measurements were taken by a group of Sports Sciences PhD students who were previously trained to correctly collect the data for each test.

### Anthropometric characteristics

Children’s stature was measured to the nearest 0.5 cm on a standardized wall-mounted height board, and body mass (BM) was measured to the nearest 0.1 kg with a calibrated manual weighing scale (Seca 709, Germany). Body mass index (BMI) was calculated as BM (kg) · stature^-2^ (m). Children were considered OW or OB based on BMI/age-specific curves when their BMI was greater than or equal to the international cut-off point corresponding to the centile curve that passes through either the BMI curve for 25 or 30 kg · m^-2^, respectively, at 18 years of age [26].

### Physical fitness

To obtain a representative status of children’s physical fitness, 6 of the several physical fitness tests suitable for the selected age group [27,28] were considered. Priority was given to the accuracy of measurements, which were taken by a group of trained sports scientists within a short period of time in all the schools involved in the study. The 6 tests selected were administered to children on 6 different weekdays, during their physical education classes, avoiding each test being influenced by the results of the previous test. The following tests, which were easy to perform in all the school environments, were chosen:

#### Aerobic capacity

Aerobic capacity was evaluated by the Léger test [29], which has confirmed validity and reliability in children [30–32]. The test involved running continuously between two points that were 20 metres apart. The runs were synchronized with a pre-recorded audio tape, which played beeps at set intervals. The interval of beeps was calculated to obtain a speed of 8.5 km· h^-1^ at the start, and to increase by 0.5 km· h^-1^ at each level thereafter. As the test proceeded, the interval between each successive beep decreased, forcing children to increase their speed over the course of the test until it was impossible to keep in sync with the recording. When the children being tested did not reach the final point in time, the last level completed was recorded as their final score.

#### Agility

Whole body agility was evaluated by a shuttle test (10 x 5 m) [17,18,33,34]. The reliability and validity of shuttle tests have been previously examined in children [35]. Two lines, 5 metres apart, were identified using cones. On the signal "ready", children were instructed to place their feet behind the starting line. Then, on the signal "go!", they sprinted to the opposite line, passed it with both feet, ran back to the starting point, and repeated the task, without a rest. Children were asked to repeat the track between the two lines 10 times, in order to run 50 metres in total. Two trials were performed, and the shortest time needed to complete the test was recorded in seconds.

#### Muscular power of the lower limb

Lower limb explosive power was evaluated by a long jump test [17,18,20,21,23,25,33,34,36,37]. The validity and reliability of jump tests have been previously evaluated in children by several authors [31,32,38]. Each child jumped for distance from a standstill. During the performance of the jumps, children were asked to bend their knees with their arms in front of them, parallel to the ground, then to swing both arms, push off vigorously and jump as far as possible, trying to land with their feet together and to stay upright. The test was performed three times and scored in centimetres. The longest jump length was recorded.

#### Muscular power of the arms

Upper body power was evaluated by a frontal throw of a basketball (0.5 kg) [39], which has been defined as being a valid and reliable test [40]. Children were asked to sit on the ground with their legs apart and their back leaning against the wall, facing the direction where the ball was to be thrown. The ball was held with two hands and brought close to the body at the chest level, then vigorously thrown forward as far as possible, maintaining wall contact. The longest throw of three, as measured by the distance between the wall and the first contact point of the ball to the ground, was recorded in centimetres.

#### Flexibility

Hip and low back flexibility were evaluated by the Sit & Reach test [17,18,20,21,23,25,33,34], whose reliability and validity have been previously assessed in the literature [32,41]. In a seated position with their knees extended and their feet placed firmly against a vertical support, children reached forward along the measuring line as far as possible with their arms at the same level. The distance reached by their hands to the nearest centimetre was recorded as the score, using the level of the feet as zero, so that any measure that did not reach the toes was considered negative and any measure beyond the toes was considered positive.

#### Static balance

The standing balance test was used in previous studies to reliably [32] evaluate balance capacity in children [25,28]. Children removed their shoes and placed their hands on their hips. While balancing on the preferred leg, the free leg was flexed at the knee, and the foot was held close to the buttocks by the hand of the same side. Children had one minute to practice their balancing before starting the test. Then, children stood on their preferred leg for a maximum of 30 s. Time was recorded when children moved their supporting foot or when they lost contact between the heel of the non-supporting leg and their buttocks.

### Statistical analysis

To perform the analysis, the collected data were first screened for incorrect inclusions. When they were not plausible, records were excluded from the database if the correct information was unavailable. Anthropometric characteristics and all physical fitness information were expressed as the mean and standard deviation (SD) or standard error (SE) in the graphs and stratified by sex and age and by sex, age and BMI categories, respectively. The effects of sex, age and BMI categories and the interaction among these variables on anthropometric characteristics and physical fitness were tested using two-way analysis of variance (ANOVA) after evaluating the homogeneity of variance with Levene’s test. When significant differences were found, a Bonferroni post hoc test was evaluated implementing multiple comparisons to detect which variable means were significantly different from each other. Then, a simple linear regression equation was calculated for each sex and for each physical fitness parameter (including 6 to 11 age) to evaluate the difference between each BMI category. We compared the regression coefficients (β) of NW with those of other BMI categories (individually for males and females) to test the null hypothesis H_0_: β_NW_ = β_OB/NW/UW_ and to evaluate whether the dimension of a regression coefficient should be larger for one group than for another. All statistical analyses were performed by SAS, Release 9.4 (SAS Institute, Cary, NC, USA), with a significance set at p<0.05.

## Results

The initial selected sample (n: 30472) was screened for any incorrect inclusions and 41 records were deleted from the original database. Table 1 shows the anthropometric characteristics of the 30431 children involved in the study. Stature and body mass (BM) increased significantly from age class 6 to age class 11 (by a mean of +0.05 m and +3.3 kg per year in both girls and boys) and differed significantly between girls and boys (p<0.001). In addition, BMI increased significantly with age (by a mean of +0.48 and +0.52 kg·m^-2^ per year in girls and boys, respectively) and differed significantly between girls and boys (p<0.002), except for age classes 6 and 7.

**Table 1:** Anthropometric characteristics of children (n: 30431) stratified by sex (girls and boys) and age class (6–11 years old).

	Girls (n:14645)						Boys (n:15786)						P		
	6 years (n:845)	7 years (n:3161)	8 years (n:3176)	9 years (n:2914)	10 years (n:2690)	11 years (n:1859)	6 years (n:868)	7 years (n:3377)	8 years (n:3303)	9 years (n:3228)	10 years (n:2929)	11 years (n:2081)	Sex	Age	S X A
Stature (m)	1.19±0.05	1.21±0.06	1.28±0.06	1.33±0.06	1.39±0.07	1.44±0.07	1.17±0.06	1.21±0.06	1.26±0.06	1.32±0.07	1.38±0.07	1.44±0.07	<.001	<.001	<.001
BM (kg)	22.6±4.0	24.1±4.6	27.3±5.6	31.1±6.9	34.7±7.7	38.9±8.8	23.1±3.7	24.5±4.5	27.7±5.4	31.7±6.8	35.4±7.6	39.4±8.6	<.001	<.001	0.757
BMI (kg·m^-2^)	16.3±2.2	16.5±2.3	17.0±2.6	17.7±3.0	18.1±3.1	18.7±3.3	16.4±2.0	16.6±2.2	17.0±2.5	17.8±2.8	18.3±3.0	19.0±3.3	0.002	<.001	0.056

All values are means ± SD. Significant according to a generalized linear model of the main effects of Sex (Girls vs Boys), Age, and Sex × Age interaction (S × A).

Table 2 shows the prevalence of underweight (UW; 7.5% *vs* 6.1%), normal-weight (NW; 67.4% *vs* 69.6%), overweight (OW; 19.2% *vs* 18.4%) and obesity (OB; 5.9% *vs* 5.8%) in girls and boys, respectively, grouped by age class. Significant differences (p<0.05) in weight status prevalence were found between girls and boys of all ages apart from those who were 6 years old (p = 0.607). Additionally, significant differences were found in UW, NW, OW and OB prevalence between age groups in girls and boys (p = 0.001, for both sexes).

**Table 2:** Underweight (UW), normal-weight (NW), overweight (OW) and obesity (OB) prevalence in girls and boys of each age class (6–11 years old).

	Girls (n:14645)							Boys (n:15786)						
	6 years (n:845)	7 years (n:3161)	8 years (n:3176)	9 years (n:2914)	10 years (n:2690)	11 years (n:1859)	Total	6 years (n:868)	7 years (n:3377)	8 years (n:3303)	9 years (n:3228)	10 years (n:2929)	11 years (n:2081)	Total
UW (%)	7.3	6.5	7.2	7.3	8.1	9.2	7.5	6.9	6.3	7.2	5.4	5.5	6.1	6.1
NW (%)	66.8	69.4	67.3	65.7	67.6	67.0	67.4	69.7	72.9	70.6	67.9	69.0	66.6	9.7
OW (%)	19.8	17.6	19.2	20.7	19.3	19.4	19.2	18.1	15.5	16.6	19.7	19.9	22.0	18.4
OB (%)	6.2	6.5	6.3	6.4	5.1	4.5	5.9	5.3	5.4	5.7	7.0	5.5	5.4	5.8

### Children’s physical fitness

#### Aerobic capacity

Girls completed fewer levels than boys in the Léger test at each age class (-0.2, -0.4, -0.5, -0.9 and -0.4 levels for ages 6 to 11 years old, respectively, p<0.011), and older children completed more levels than younger children (by a mean of +0.3 levels each year, in both girls and boys). This performance improvement by age followed a diminishing pattern. Between class ages 6 and 7, the level reached by girls and boys increased by +0.5 and +0.7 levels, respectively, while between class ages 10 and 11, it increased by +0.1 and +0.4 levels, respectively (Table 3).

**Table 3:** Physical fitness scores of children (n: 30431) stratified by sex (girls and boys) and age class (6–11 years old).

	Girls (n:14645)						Boys (n:15786)						P		
	6 years (n:845)	7 years (n:3161)	8 years (n:3176)	9 years (n:2914)	10 years (n:2690)	11 years (n:1859)	6 years (n:868)	7 years (n:3377)	8 years (n:3303)	9 years (n:3228)	10 years (n:2929)	11 years (n:2081)	Sex	Age	S X A
Léger (n)	1.6±0.9	2.1±1.6	2.6±2.1	2.8±1.5	3.0±1.8	3.1±1.6	1.8±1.1	2.5±1.8	3.1±2.2	3.4±1.9	3.9±2.4	3.5±1.9	0.014	<.001	0.011
Agility Shuttle (s)	26.7±3.7	26.0±4.0	25.3±5.7	24.1±5.1	23.4±4.1	23.0±4.0	26.2±4.0	25.4±4.4	24.3±5.2	23.7±6.0	22.7±4.3	22.2±3.5	<.001	<.001	0.012
Long jump (cm)	94.4±17.4	97.0±18.4	106.6±18.8	115.3±19.3	122.8±20.6	128.8±20.6	101.0±19.2	103.6±19.3	113.8±20.1	122.9±20.5	130.7±21.5	137.7±22.3	<.001	<.001	0.006
Frontal throw (cm)	221.6±52.4	235.9±54.8	282.2±59.6	326.9±67.7	366.7±72.2	405.7±72.2	239.7±56.9	254.5±61.2	309.1±66.6	359.8±74.9	406.3±76.0	448.3±78.7	<.001	<.001	0.024
Sit & Reach (cm)	3.0±6.2	2.6±6.8	2.5±7.2	2.4±7.8	2.3±8.1	2.3±8.6	0.2±6.2	-0.9±6.5	-1.9±7.4	-2.9±7.5	-3.0±7.9	-3.6±8.4	<.001	<.001	0.027
Standing balance (s)	15.9±9.9	17.8±8.6	21.1±8.3	23.3±7.6	24.8±6.8	25.5±6.0	13.0±9.2	14.8±8.5	18.0±8.8	20.5±8.4	22.8±7.8	23.6±7.2	<.001	<.001	0.015

All values are means ± SD. Significant according to a generalized linear model of the main effects of Sex (Girls vs Boys), Age, and Sex × Age interaction (S × A).

**Fig 1:**
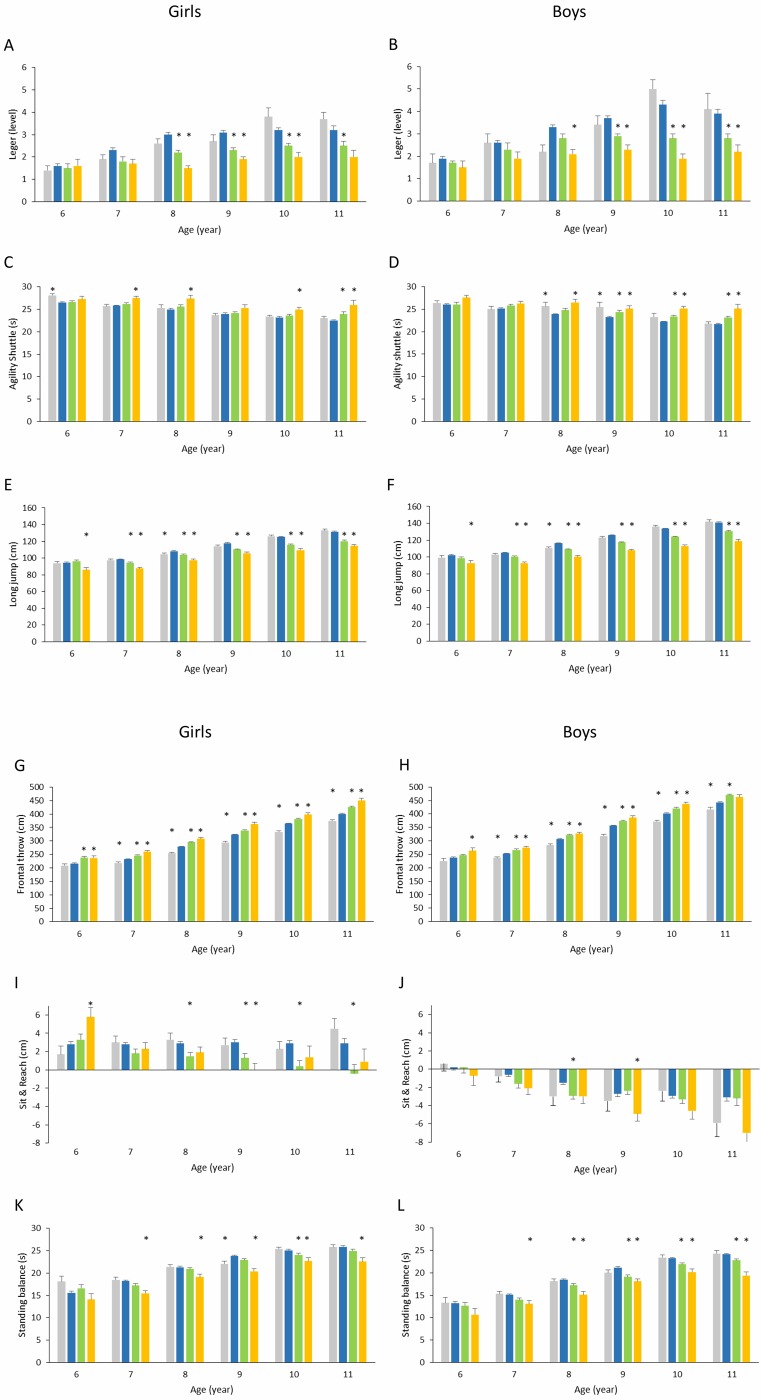
a. Children’s physical fitness (Léger test: A, B; Agility shuttle test: C, D; Long jump test: E, F) reported as a function of BMI category and age class. Girls: A, C, E; Boys: B, D, F. Underweight, ■; normal weight, ■; overweight, ■; and obese, ■. All values are means ± SE. *Significantly different relative to scores for normal weight children (p<0.05). b. Children’s physical fitness (frontal throw test: G, H; Sit & Reach test: I, J; standing balance test: K, L) reported as a function of BMI category and age class. Girls: G, I, K; Boys: H, J, L. Underweight, ■; normal weight, ■; overweight, ■; and obese, ■. All values are means ± SE. *Significantly different relative to scores for normal weight children (p<0.05).

OB girls and OB boys performed worse on the Léger test than their NW counterparts (Fig 1a, 1aA and 1aB). In particular, significantly lower levels were reached by 8-, 9- and 10-year-old OB girls than by NW girls of the same ages (-50.0, -38.7 and -37.5%, respectively, p<0.001) and by 8-, 9-, 10- and 11-year-old OB boys than by NW boys (-36.4, -37.8, -55.8 and -43.6%, respectively, p<0.001). Additionally, OW girls completed lower levels than NW girls at 8, 9, 10 and 11 years old (-26.7, -25.8, -21.9 and -21.9%, respectively, p<0.001), and OW boys reached lower levels than NW boys at 9, 10 and 11 years old (-21.6, -34.9 and -28.2%, respectively, p<0.001).

**Fig 2:**
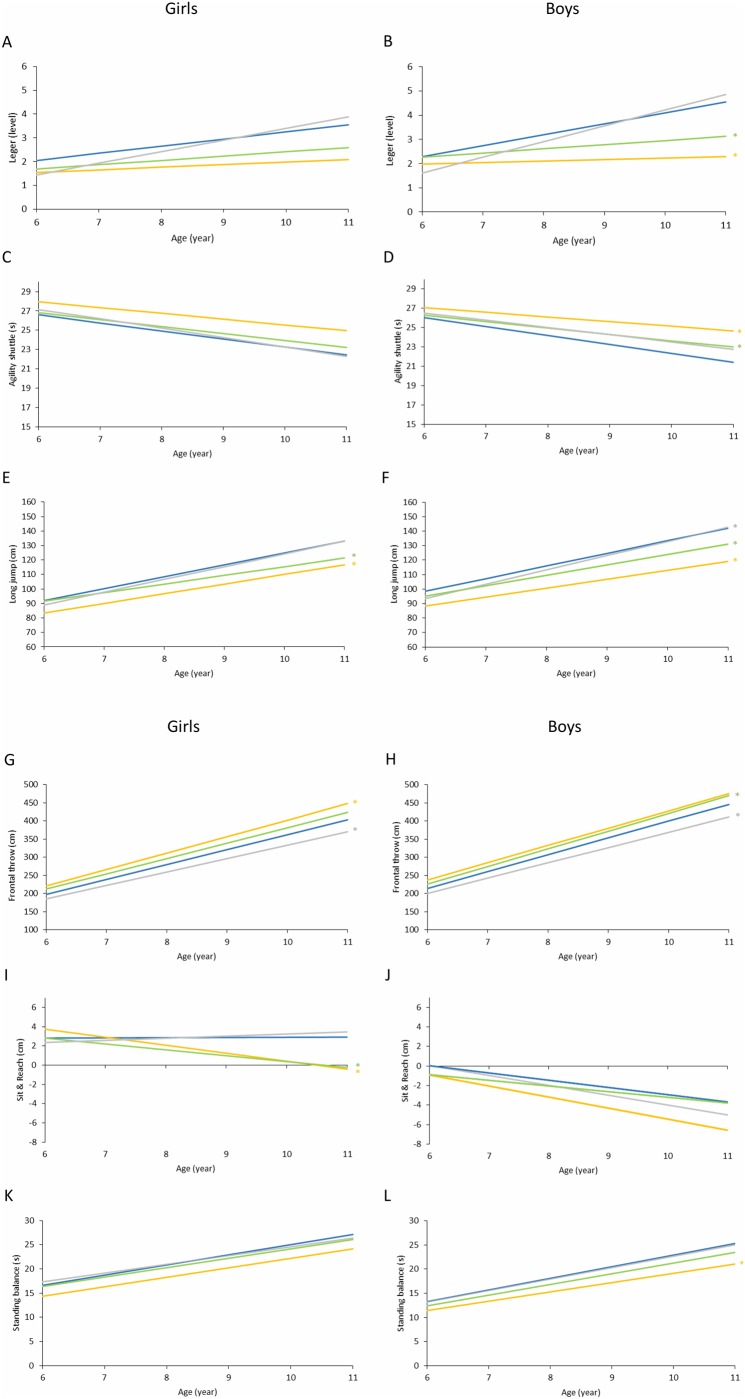
a. Regression models considering changes in children’s physical fitness (Léger test: A, B; Agility shuttle test: C, D; Long jump test: E, F) reported as a function of BMI categor*y* and age class. Girls: A, C, E; Boys: B, D, F. Underweight, ----; normal weight, ----; overweight, ----; and obese, ----. *Significantly different slope relative to that for normal weight children (p<0.05). b. Regression models considering changes in children’s physical fitness (frontal throw test: G, H; Sit & Reach test: I, J; standing balance test: K, L) reported as a function of BMI category and age class. Girls: G, I, K; Boys: H, J, L. Underweight, ----; normal weight, ----; overweight, ----; and obese, ----. *Significantly different slope relative to that for normal-weight children (p<0.05).

The discrepancy in aerobic capacity among OB, OW and NW children increased at higher age classes relative to lower classes (Fig 2a, 2aA and 2aB). Nevertheless, the slopes of the linear regression models calculated for OB and OW girls were not significantly different from those for NW girls, whereas the slopes calculated for OB and OW boys were significantly lower than those for NW boys (p = 0.009 and p<0.001, respectively).

#### Agility shuttle

Girls needed more time than boys to complete the shuttle test at each age class (+0.5, +0.6, +1.0, +0.4, +0.7 and +0.8 s from 6 to 11 years, respectively, p<0.012). Moreover, agility improved progressively from age class 6 to age class 11 in children of either sex (by a mean of -0.7 s in girls and -0.8 s in boys each year).

The amount of time needed to complete the test was significantly longer in OB girls and boys than in NW girls and boys (Fig 1a, 1aC and 1aD). In particular, OB girls aged 7, 8, 10 and 11 needed more time than their NW peers (+6.6, +9.6, +7.8 and +15.6%, respectively, p<0.001), as did OB boys aged 8 to 11 (+10.9, +8.2, +13.5 and +15.7%, respectively, p<0.001). Similarly, 11-year-old OW girls took longer to complete the test than NW girls (+6.2%, p = 0.0231), as did 9-, 10- and 11-year-old OW boys compared to NW boys (+5.2, +5.4 and +6.5%, respectively, p< 0.001). On the other hand, UW girls showed worse agility capacity than NW girls at 6 years old (+6.2%, p = 0.023), and UW boys showed worse agility capacity than NW boys at 8 and 9 years old (+7.5% and +9.9%, respectively, p<0.033).

The agility gap between OB, OW and NW children increased in older children compared to younger children (Fig 2a, 2aC and 2aD). No significant slope differences were found between NW girls and other BMI categories. However, the slopes calculated for OB and OW boys were significantly greater than the slope calculated for NW boys (p = 0.006 and p = 0.008, respectively).

#### Muscular power of the lower limb

Girls jumps were shorter than those of boys for each age class (-6.6 cm at 6 and 7 years old, -7.2 cm at 8 years old, -7.6 cm at 9 years old, -7.9 cm at 10 years old and -8.9 cm at 11 years old, p<0.006). Muscular power of the lower limb was considerably greater in older children than in younger children (mean +6.9 cm in girls, and +7.3 cm in boys, per year). The peak improvement was observed between 7 and 8 years (+9.6 and +10.2 cm in girls and boys, respectively).

The recorded jump distance was shorter in OB and OW children than in NW and UW children for either sex (Fig 1a, 1aE and 1aF). In particular, OB girls jumped a significantly shorter distance than their NW counterparts at each age class (-9.3, -10.9, -10.0, -10.3, -12.7 and -13%, at 6, 7, 8, 9, 10 and 11 years old respectively, p<0.001), as did OB boys (-9.5, -11.6, -13.8, -14.4, -15.6 and -15.9%, respectively, p<0.001). OW girls jumped a shorter distance than NW girls at 7, 8, 9, 10 and 11 years old (-4.0, -4.1, -6.4, -7.5 and -8.6%, respectively, p <0.001), as did OW boys (-4.3, -6.2, -6.8, -7.3 and -7.6%, respectively, p <0.001). On the other hand, 8-year-old UW girls and boys jumped a significantly shorter distance than their NW counterparts (-3.3%, p = 0.033 and -4.9%, p<0.001, respectively).

Lower limb muscular power disparities among children of differing BMI categories were found to be wider in higher age classes than in lower age classes (Fig 2a, 2aE and 2aF). The slopes of the regression models calculated for OB and OW children were significantly lower than the slope calculated for NW children of either sex (p<0.001 and p = 0.002 for OB and OW girls, respectively, and p<0.001 for OB and OW boys). On the other hand, the regression slope for UW boys was significantly higher than that of NW boys (p = 0.015).

#### Muscular power of the arms

Girls scored lower than boys on the frontal throw test at each age class (-18.1, -18.6, -26.9, -32.9, -39.6, -42.6 cm from 6 to 11 years, respectively, p<0.024). Muscular power of the upper limb increased from age class 6 to age class 11 in both girls and boys (by a mean of +36.8 and +41.7 cm per year, respectively). A peak was observed between 7 and 8 years old (+46.3 and +54.6 cm in girls and boys, respectively).

The distance covered by the thrown ball increased gradually from lower to higher BMI categories (Fig 1b, 1bG and 1bH). In particular, OB girls threw the ball significantly farther than NW girls at 6, 7, 8, 9, 10 and 11 years old (+9.3, +12.3, +10.5, +12.3, +9.3 and +12.3%, respectively, p<0.001), and OB boys threw the ball significantly farther than NW boys at 6, 7, 8, 9 and 10 (+10.9, +9.3, +6.3, +8.5 and +8.8%, p<0.001). Similarly, OW girls threw farther than NW girls at 6, 7, 8, 9, 10 and 11 years old (+9.9, +5.8, +5.7, +5.0, +4.8 and +6.2%, respectively, p<0.001), and OW boys threw farther than NW boys at 7, 8, 9, 10 and 11 (+5.9, +4.7, +5.1, +4.6 and +6.2%, respectively, p <0.001). Finally, UW girls scored significantly lower than NW girls at 7, 8, 9, 10 and 11 years old (-6.4, -8.9, -8.8, -8.5 and -6.6%, respectively, p <0.001), as did UW boys (-5.7, -7.5, -10.6, -7.5 and -5.7%, respectively, p <0.001).

The gap in muscular power of the arms among children belonging to different weight status categories was greater in older children than in younger children (Fig 2b, 2bG and 2bH). OB girls and OW boys showed a significantly greater linear regression slope than NW girls and boys, respectively (p = 0.005 and p = 0.011). In contrast, UW girls and boys showed significantly smaller slopes than NW girls and boys (p = 0.003 and p = 0.011, respectively).

#### Flexibility

Girls obtained higher Sit & Reach scores than boys at each age class (+2.8, +3.5, +4.4, +5.3, +5.3, +5.9 cm from 6 to 11 years, respectively, p<0.027). Moreover, there was no significant difference in flexibility scores obtained by girls of differing age groups; on the other hand, scores were found to be significantly lower for older boys than for younger boys (by a mean of -0.8 cm each year).

Flexibility was partially influenced by BMI status (Fig 1b, 1bI and 1bJ). OB girls showed greater flexibility capability at 6 years old and lower capability at 9 years old (+107.1 and -100.0%, p<0.004) than NW girls, while OW girls were significantly less flexible than NW girls at 8, 9, 10 and 11 years old (-48.3, -56.7, -86.2 and -113.8%, respectively, p<0.005). On the other hand, BMI was found to have less impact on the flexibility of boys: only 9-year-old OB boys and 8-year-old OW boys performed significantly worse than their NW peers (-81.5%, p = 0.021 and -93.3%, p = 0.045, respectively).

Additionally, it was observed that the flexibility gap between children of differing BMI categories was greater in older children than in younger children, especially in girls (Fig 2b, 2bI and 2bJ). Indeed, only the slopes calculated for OB and OW girls were significantly different from those of their NW peers (p = 0.002 and p<0.001, respectively).

#### Static balance

Balance capacity was found to be better in girls than in boys at each age class (+2.9, +3.0, +3.1, +2.8, +2.0, +1.9 s from 6 to 11 years old, respectively, p<0.015) and to increase by age in both sexes (by a mean of +1.9 s in girls and + 2.1 s in boys each year). A peak was observed between 7 and 8 years old (+3.3 and +3.2 s in girls and boys, respectively).

Significant differences in standing balance scores were observed among BMI categories at most age classes, except for age class 6 (Fig 1b, 1bK and 1bM). With respect to NW girls, worse balance capacity was shown at 7, 8, 9, 10 and 11 years old by OB girls (-14.8, -10.3, -14.3, -9.6 and -12.7%, respectively, p<0.001), at 10 years old by OW girls (-4.0%, p = 0.034), and at 9 years old by UW girls (-7.1%, p = 0.008). Similarly, at 7, 8, 9, 10 and 11 years old, OB boys maintained their balance for a significantly shorter time than NW boys (-13.2, -18.4, -14.6, -13.3 and -19.8%, respectively, p<0.001), as did OW boys at 8, 9, 10, and 11 years old (-7.0, -9.9, -6.0 and -5.8%, respectively, p<0.001).

Fig 2b, 2bK and 2bL show the regression models considering changes in the balance scores of girls and boys as a function of BMI category and age class. The only statistically significant slope difference was found between OB and NW boys (p = 0.019).

## Discussion

The main results showed that in the present sample of Italian prepubertal children, 1) anthropometric characteristics and physical fitness differed significantly between girls and boys; 2) OB and OW status negatively affected aerobic capacity, agility, lower limb power and balance but 3) positively affected upper limb power; 4) underweight negatively affected upper limb power; and finally, 5) BMI effect on physical fitness increased over the years.

Both the anthropometric characteristics and physical fitness of prepubertal children are affected by sex and age. As has already been observed in the literature, sex-related differences in anthropometric characteristics and physical fitness increase in older children, particularly after 12 years of age [34,42,43]. Actually, according to some authors [24,33,44], female improvement in physical fitness (particularly in strength) plateaus at approximately 12 years old, marking the emergence of the gender gap. However, as confirmed by the present study, sex-related differences could also be detected prior to the pubertal stage [18,21,37,45,46]. In agreement with several previous studies assessing children’s physical fitness through field-based tests similar to those used in the present study, it was observed that boys perform better than girls in terms of cardiovascular fitness [18,33,34,37,44,46,47], muscular strength of the upper and lower limb [18,21,24,33,34,37,44,46,47] and speed-agility [18,21,33,34,37,46,47]. Conversely, girls perform better in terms of flexibility [18,21,33,34,37,44,47] and balance [33,37,46]. The observed sex-related differences might be due to both environmental and biological factors. It is known that children who play sports have better physical fitness than those who do not [21]. Moreover, physical activity attendance and type of sport practised could be different in girls and boys due to motivation, social interest or peer influence, resulting in girls being generally less active than boys [48]. Regarding biological factors, although we did not collect data on body composition to confirm these assumptions, previous studies showed that girls have a significantly greater percentage of fat mass and less fat free mass than boys [49] and that, during growth, the fat free mass of males increases at a faster rate than that of females, especially during puberty [50]. Consequently, these environmental and biological differences might have led to better physical fitness and muscular strength performances in boys than in girls, especially at older ages.

Additionally, consistent with previous studies regarding European prepubertal children [18,24,25,33,34,37,45,46], a general performance improvement by age was detected in each fitness parameter, except for flexibility. In this regard, it is known that gross motor coordination improves from childhood to puberty [44] even if inter-individual variation is still a major feature among typically developing young children [51]. Concerning flexibility, the present study revealed a significant decrement in boys’ performance from younger to older age, as was reported by Gonzales et al. [18]. However, some authors [37] have found a non-significant variation in the flexibility scores of both sexes by age, while others have found an improvement with age in girls and an improvement after puberty in boys [34]. Such inconsistencies might be due to individual variation or the type of physical activity practised by the specific sample of children considered.

Not only sex and age can affect children’s physical fitness. Body mass and body composition account for a substantial portion of the variation in performance during childhood [21,22]. Consistent with previous literature findings, the present study detected statistically significant differences between various physical fitness scores achieved by OB, OW, NW and UW children. Specifically, it is known that OW and OB children perform worse than NW children in weight bearing activities, as was the case in the Leger, shuttle and jump tests [16–25]. In the present study, this trend was found to be similar between sexes and more prominent in older children than in younger children, emphasizing the need to promote healthier lifestyles from an early age. Moreover, OW *vs* NW differences were less frequently significant than OB *vs* NW differences, and OW children performed better than OB children in weight bearing activities, as has been reported in the literature [17]. This could be due to the quantitative role of fat mass, which behaves as an inert load limiting physical movement, physical activity attendance [52] and proper motor skills development in OB children more than in OW children. On the other hand, the increased mechanical work needed to lift the body off the ground in everyday-life activities could have a positive influence on absolute muscle strength, increasing fat free mass (FFM) more in OB children than in their NW counterparts [53,54]. A higher FFM, which is known to correlate with strength parameters in obese subjects [55], might be the reason why better throwing scores were obtained by the OB and OW children in the present and in previous studies [18,20,21,24] than by NW children. In contrast, some authors [23,25] have found comparable strength of the upper limb in OB, OW and NW children. These conflicting results might be explained by different throwing protocols, body composition or physical activity practised by children. Additionally, our results confirmed that being OB or OW has a negative effect on static balance capability [25] and, in older girls in particular, on flexibility [19–21,23,25]. In fact, the flexibility scores achieved by OW and OB girls worsened dramatically with age but remained stable in NW and UW girls. Finally, although the relationships between UW children and their physical fitness have been less studied in the literature, some authors noticed that UW children perform similarly to NW children apart from in upper [16,18,23,24] and lower limb strength [16,23], in which they obtained lower results. In the present study, UW children performed considerably worse than NW children in upper limb strength, while, regarding the long jump test, significantly lower scores were observed for the UW children than for the NW children exclusively at 8 years old. Other significant worse results were obtained by UW children than by NW children on the agility shuttle test. These findings suggest that not only obesity but also leanness could have an impact on children’s physical fitness. Indeed, although being UW at 6 years old might not have major fitness consequences, it could lead to more consistent impairments at an older age, if not corrected.

Among participants taking part in the present study, we noticed that the prevalence of OW and OB (24.6%) was lower than in previous Italian studies [2,21,56,57], which is in line with the prevalence observed in northern Italy in 2016 [3]. Comparing our results with data regarding children of the same age in the same area, the general trend of OB, OW and UW appears to be slightly reducing [25]. This trend was also found in another Italian region in 2015 [56]. Nevertheless, the OB and OW prevalence in children still remains higher than the mean value observed in Europe between 2007 and 2010 [4] and needs to be reduced promptly.

Because of the alarming prevalence of OW and OB children and the low cardiorespiratory fitness levels in southern European youth [34], it is important to identify, at an early age, children who are likely to develop low levels of physical fitness to adopt appropriate measures to counter these deficiencies. Our results showed that BMI has a significant impact on physical fitness capacity and development in prepubertal children. Therefore, interventions should be primarily addressed to children with non-optimal weight status. Although some limitations must be considered when interpreting the findings of this study, such as the cross-sectional design and the limited geographical region considered, the large sample size allowed us to obtain informative data on Italian prepubertal children’s physical fitness and weight status. In conclusion, the study provided evidence that sex, age, and BMI-related differences in physical fitness could be detected before puberty, and that preventing childhood overweight, obesity, and underweight in early life is extremely important to promote children’s health and optimal physical fitness development, suggesting that the earlier the intervention is implemented, the more effective it will be.
